# Gastric cancer, what’s new from International Gastric Cancer Congress (IGCC) in Amsterdam: a scientific report from Salerno 2025 conference

**DOI:** 10.3332/ecancer.2026.2083

**Published:** 2026-03-09

**Authors:** Dario Cattel, Pasquale Valerio Albano, Rosario De Feo, Stefano Pepe, Alessandro Puzziello

**Affiliations:** 1 General Surgery Unit, AOU San Giovanni di Dio e Ruggi d’Aragona, University of Salerno, Largo Città di Ippocrate, 84131 Salerno, Italy; 2 General and Emergency Surgery, 'Santa Maria Incoronata dell'Olmo' Civil Hospital, University of Salerno, Cava de' Tirreni, V. Enrico de Marinis, 84013 Salerno, Italy; 3 Oncology Unit, Department of Medicine, Surgery and Dentistry, University of Salerno, 84081 Baronissi, Italy; a https://orcid.org/0009-0006-4988-0733; b https://orcid.org/0009-0001-1399-0344; c https://orcid.org/0009-0009-9573-5096; d https://orcid.org/0000-0001-6280-8142; e https://orcid.org/0000-0002-1970-7386

**Keywords:** gastric cancer, multidisciplinary management, gastrectomy, oncological networks, personalized medicine, surgical oncology

## Abstract

This paper presents a critical synthesis of the national conference “Gastric Cancer at Ruggi d’Aragona”, held in Salerno, Italy, in June 2025. The event gathered over 200 specialists to discuss recent advances in the multidisciplinary management of gastric cancer. Key topics included precision surgery, integration of imaging and molecular profiling, innovations in minimally invasive and robotic techniques, and emerging strategies such as conversion therapy and neoadjuvant immunotherapy. Insights from the 16th International Gastric Cancer Congress highlighted global trends, including the rise of targeted and immunologic approaches and the expansion of collaborative networks. The analysis identifies gaps between technological potential and clinical implementation and outlines future priorities for research and healthcare organization. This report aims to inform clinical practice and foster interdisciplinary reflection on the evolving management of gastric cancer.

## Toward an integrated and personalised surgical management of gastric cancer: reflections from a national conference

On 20 June 2025, the national conference *‘Gastric Cancer at Ruggi d’Aragona’* was held at the Aula Scozia of the University Hospital ‘San Giovanni di Dio e Ruggi d’Aragona’ in Salerno, Italy. Rather than providing a descriptive account of the event, this article offers a critical synthesis of the clinical and surgical insights that emerged, highlighting their implications for clinical practice and future research.

## Methodological framework

The event, accredited by the Italian Ministry of Health under the National Continuing Medical Education program, brought together over 200 healthcare professionals involved in the multidisciplinary management of gastric cancer. Speakers and topics were selected to represent leading national referral centers and to reflect recent scientific advances in gastric oncology, with a focus on technological innovation, integrated care pathways (PDTA) and patient-centered models of care.

## Evolving roles within the multidisciplinary team

The opening session underscored the evolving roles of healthcare professionals in multidisciplinary oncology teams, particularly the growing involvement of general practitioners and case managers in early diagnosis and coordinated care. Presentations by Dr. Sirica and Dr. Petruzzi highlighted the benefits of integrating these roles within regional oncology networks, such as the Campania Oncological Network, to facilitate timely access to diagnosis and treatment. The contribution of patient associations, illustrated by A. Guidi, further emphasised a shift toward participatory healthcare, which is increasingly vital in long-term cancer care.

A noteworthy contribution by Dr. Annechiarico addressed therapeutic management in elderly and frail patients – an underrepresented subgroup in clinical guidelines and randomised trials. The scarcity of real-world outcome data in this population underscores the need for comparative studies on personalised versus standard treatment strategies.

## From imaging and pathology to precision surgery

The critical role of imaging and pathology in preoperative planning was clearly emphasised. Dr. Erra presented reproducible, imaging-based criteria to assess resectability, though these require prospective validation. In parallel, the presentations by Dr. Zeppa and Dr. Saragoni demonstrated how histopathologic and molecular profiling are increasingly informing prognosis and guiding therapeutic decisions, particularly in the neoadjuvant setting.

These contributions reflect a paradigm shift: surgical decision-making is no longer solely guided by tumour staging but by an integrated assessment of radiological, biological and systemic factors. However, the lack of a standardised clinical model to synthesise these parameters into routine practice remains an open challenge.

## Surgical techniques and outcomes: navigating innovation

The second session focused on emerging surgical technologies in gastrectomy. Although robotic surgery, as described by Dr. Giacopuzzi, offers notable advantages in terms of precision and ergonomics, long-term oncologic outcomes remain underexplored. As emphasised by Drs. Milone and Baiocchi, the choice between open, laparoscopic and robotic approaches must be individualised based on tumour location, disease stage and institutional expertise. Dr. Pirozzi highlighted the importance of achieving' textbook outcomes’ as a marker of surgical quality, while also pointing out the lack of consistent definitions and adoption across centers.

The session concluded with a keynote lecture by Dr. Giacopuzzi, summarising major advances presented at the 16th International Gastric Cancer Congress (IGCC), held in Amsterdam in 2025. His analysis illustrated the rapidly evolving therapeutic landscape in gastric cancer, shaped by accelerated clinical research, the integration of advanced technologies and a growing emphasis on global collaboration.

Among the most promising developments was the use of ***immunotherapy in the neoadjuvant setting***. A Phase II trial reported encouraging results with ***serplulimab (HANSIZHUANG), an anti‑PD‑1 agent,*** in combination with chemoradiotherapy for resectable esophagogastric junction adenocarcinoma. The study demonstrated substantial potential for achieving radical resection, suggesting a possible shift in the treatment paradigm for this subset of patients [4].

Further data highlighted the potential of ***bispecific immunotherapeutic agents***, including ***AK104***, which targets PD‑1 and CTLA‑4. Trials in China have shown promising results in neoadjuvant contexts [6,7,8]. Likewise, ***Claudin18.2-targeted monoclonal antibodies*** showed efficacy in patients with high expression levels, especially when combined with chemotherapy or other immunotherapies – paving the way for new directions in precision oncology.

In the surgical domain, the IGCC confirmed the growing role of ***minimally invasive techniques***. The CLASS‑01 and CLASS‑02 trials demonstrated the non-inferiority of laparoscopic distal and total gastrectomy, respectively, compared to open surgery in terms of oncological safety and postoperative recovery. Preliminary results from the CLASS‑03 study also showed that ***robot-assisted surgery using the da Vinci system*** may further enhance surgical precision and improve postoperative quality of life [3].

Regarding ***peritoneal recurrence prevention***, preliminary studies on ***hyperthermic intraperitoneal chemotherapy (HIPEC)*** in high-risk patients indicated a potential benefit in disease-free survival, although larger trials are needed to validate these findings.

In the metastatic setting (Stage IV), the ***MetaGastro*** trial introduced new chemotherapy regimens now being incorporated into clinical practice. Additionally, the ***PECORINO trial*** compared FLOT and XELOX regimens in terms of perioperative toxicity and provided new insights into personalised treatment selection based on tolerability profiles [11].

Dr. Giacopuzzi also emphasised the global dimension of the IGCC 2025, which attracted approximately 950 participants from 50 countries, with 980 abstracts submitted from 39 nations. A wide array of satellite activities – including interactive sessions, informal tours and networking dinners – helped foster an international scientific community and laid the groundwork for future collaborative projects.

In summary, IGCC 2025 highlighted five key trends: the rise of neoadjuvant immunotherapy [4,5,9], the expansion of minimally invasive and robotic surgery [1,2,3], the potential of HIPEC as a preventive strategy, the evolution of systemic therapies for metastatic disease and the critical role of international networking in driving innovation in gastric oncology.

## Metastatic disease and emerging therapeutic perspectives

The final session addressed complex and evolving topics such as the management of metastatic disease and postoperative complications. Dr. Francesca Steccanella, Scientific Director of the event, delivered a comprehensive overview of the evolving concept of ***conversion therapy***, revisiting it a decade after the publication of Yoshida’s criteria [10].

In gastric cancer, conversion therapy refers to a therapeutic strategy applied to patients with initially unresectable or advanced disease, with the aim of enabling curative-intent surgery following systemic treatment. This approach typically involves chemotherapy or targeted therapies aimed at tumour reduction or disease control, thereby allowing for subsequent resection that was not feasible at initial diagnosis.

Dr. Steccanella emphasised that conversion therapy is intended for carefully selected patients – particularly those with limited metastatic burden (e.g., lymphatic, hepatic or peritoneal) – who exhibit a favourable response to systemic therapy. Positioned between neoadjuvant therapy, which is administered to operable tumours to improve surgical outcomes and palliative therapy, which focuses on symptom control in advanced unresectable disease, conversion therapy seeks to redefine the therapeutic trajectory in responsive cases.

The discussion underscored the importance of rigorous patient selection, dynamic tumour response assessment and multidisciplinary decision-making in determining optimal timing for surgical intervention. While preliminary data are promising in terms of survival benefit compared to palliative approaches alone, Dr. Steccanella noted the lack of large-scale randomised trials to establish its definitive clinical value. As such, conversion therapy remains an exciting yet investigational strategy, pending further validation before broader adoption in clinical guidelines.

The session concluded with presentations by Dr. Laurino and Dr. Zulli, who explored the roles of interventional radiology and endoscopy in managing postoperative complications – areas often underappreciated but crucial for improving long-term surgical outcomes.

## Discussion and conclusion

The conference reaffirmed the biological, clinical and organisational complexity of gastric cancer, a malignancy still associated with high morbidity and mortality. Nonetheless, it is increasingly manageable through the integration of technological innovation, centralised care pathways and multidisciplinary collaboration. The presentations highlighted a progressive shift in surgical oncology toward greater technical precision, improved selection of candidates for radical surgery and growing personalisation of treatment strategies based on tumour biology and patient-specific factors.

However, a consistent theme that emerged was the gap between the potential offered by novel technologies (surgical, pharmacologic, diagnostic and organisational) and their real-world implementation. This discrepancy reflects, in part, a lack of high-level evidence from prospective controlled studies and, in part, the variability in healthcare delivery models across regions and institutions.

In this context, several research and development priorities were identified to help bridge these gaps. These include:

- Conducting randomised comparative trials to assess the efficacy and safety of different surgical approaches (open, laparoscopic and robotic), with attention to long-term oncologic outcomes and postoperative quality of life;
- Standardising radiologic and molecular criteria for resectability and treatment response to optimise patient selection and therapeutic appropriateness;
- Implementing prospective registries and real-world data analyses focused on elderly and frail patients, who are underrepresented in clinical trials but highly prevalent in practice;
- Validating integrated care models, such as PDTA (Diagnostic-Therapeutic Care Pathways), across different regional and institutional settings to assess their impact on diagnostic timeliness, guideline adherence and survival outcomes.

Finally, the importance of continuous professional education, interdisciplinary collaboration and the establishment of national and international research networks emerged as critical drivers of innovation. More than a summary of a scientific meeting, this paper aims to contribute to a critical understanding of ongoing developments in gastric cancer management and to stimulate dialogue among clinicians, researchers and policymakers working to improve the effectiveness and equity of oncologic care.

## Conflicts of interest

The authors declare that they have no conflicts of interest related to the content of this manuscript.

## Funding

The authors declare that no funds, grants, or other support were received for the preparation of this work.
